# Using Genomic Data in Personalized Oncology Medicine: An Exploratory Expert-Elicitation Study with Narrative Regulatory Mapping of Regulations, Implementations, and Challenges in Saudi Arabia

**DOI:** 10.3390/healthcare14183029

**Published:** 2026-09-15

**Authors:** Lamees Hakami, Lamya Alnaim, Khalid Almoaikel, Jawaher Alharbi

**Affiliations:** 1 IQVIA Middle East and North Africa, Riyadh 11451, Riyadh Province, Saudi Arabia; lamees.28@outlook.com (L.H.); kalmoaikel@gmail.com (K.A.); 2 Pharmacy College, King Saud University, Riyadh 11451, Riyadh Province, Saudi Arabia; 3 King Faisal Specialist Hospital and Research Center, Riyadh 12713, Riyadh Province, Saudi Arabia; jawaherskh@gmail.com

**Keywords:** personalized oncology, cancer genes, genomic data, regulations, laws, Saudi healthcare, qualitative research, expert interviews, regulatory mapping

## Abstract

**Highlights:**

**What are the main findings?**

- We used a mixed-methods exploratory approach combining expert interviews with narrative regulatory document review to examine the current regulatory landscape, practical implementation, and challenges associated with the use of genomic data in personalized oncology medicine in Saudi Arabia.
- Through in-depth expert interviews (*n* = 4) and an analysis of existing regulations, key thematic areas were identified using qualitative thematic analysis.

**What are the implications of the main findings?**

- We report challenges of integrating genomic data into personalized oncology in Saudi Arabia, emphasizing the necessity of a national regulatory framework, clear guidelines, robust infrastructure, and strong stakeholder collaboration.
- These findings, although based on a purposively selected expert sample, provide preliminary insights that warrant further investigation with broader stakeholder engagement and quantitative validation.

**Abstract:**

Background/Objectives: Personalized medicine represents a transformative approach to healthcare in Saudi Arabia. However, a significant research gap exists regarding the specific regulatory, implementation, and practical challenges of integrating genomic data into personalized oncology care in the Saudi context. We aimed to conduct an exploratory assessment of the current regulatory landscape, practices, and challenges related to genomic data use in personalized oncology in Saudi Arabia and to identify regulatory and practical gaps, propose actionable solutions, and outline future directions for enhancing genomic integration into cancer treatment. Methods: A mixed-methods exploratory design incorporating qualitative expert interviews and a narrative regulatory review was adopted. Results: We integrated qualitative insights from four subject-matter experts with evidence from a non-systematic literature review and a narrative review of regulatory documents issued by Saudi stakeholders and international authorities. The findings identified six key themes comprising (1) the emerging role of personalized medicine in cancer care, (2) current practice and Saudi genomic initiatives, (3) regulatory and policy gaps, (4) ethical and data privacy concerns, (5) infrastructure gaps and genetic testing barriers, and (6) stakeholder collaboration and data sharing, with subthemes including healthcare providers’ skills, patient awareness, expert recommendations, future perspectives, and a descriptive regulatory comparison. However, these themes represent preliminary findings requiring further validation. Conclusions: We provide preliminary insights highlighting significant challenges in integrating genomic data into personalized oncology in Saudi Arabia, emphasizing the need for a national regulatory framework, clear guidelines, robust infrastructure, and strong stakeholder collaboration. These findings offer a foundational roadmap for future research and policy development.

## 1. Introduction

The Kingdom of Saudi Arabia faces a considerable and growing cancer burden [1,2]. According to GLOBOCAN 2022 data from the International Agency for Research on Cancer and the incidence report from the National Cancer Center (NCC) of the Saudi Health Council, an estimated 28,100 new cancer cases and 13,400 cancer-related deaths occurred in Saudi Arabia in 2022 [3]. These figures underscore the urgent need for advanced therapeutic approaches, including personalized oncology, to address the growing cancer burden in the Kingdom. Saudi Arabia continues to advance cancer care through the implementation of a new model of care, reflecting the broader ambitions of Vision 2030 to transform the healthcare system and improve population health outcomes [4].

Despite the global momentum toward personalized oncology and significant investments in genomic medicine under Saudi Vision 2030, no prior study has specifically examined the regulatory, ethical, and practical challenges associated with genomic data use for personalized oncology in the Saudi Arabian context. The existing literature focuses primarily on Western regulatory frameworks (the Food and Drug Administration [FDA] and the European Medicines Agency [EMA]) or on general genomic medicine initiatives, leaving a critical gap in understanding how these translate to the Saudi healthcare ecosystem, which operates under distinct regulatory structures (the Saudi Food and Drug Authority [SFDA], the Saudi Data and Artificial Intelligence Authority [SDAIA], and the Ministry of Health [MOH]) and cultural considerations. In this study, we address this gap by providing, to our knowledge, the first exploratory assessment of stakeholder perspectives and regulatory mapping in this domain.

Personalized medicine, defined as “matching the right drugs to the right patients” [5], is often used synonymously with precision medicine [6]. Precision medicine integrates population-level data, including big data resources, to inform diagnosis and therapy. In contrast, personalized medicine focuses on tailoring healthcare interventions to the unique genetic, clinical, and environmental profiles of individual patients.

This advanced strategy enables genome-informed drug selection, supports more accurate and effective treatment [7,8], and improves the stratification and timing of healthcare [5]. This approach is increasingly applied across various medical disciplines, with oncology being the most prominent area in which treatment decisions are often guided by the identification of disease-specific genetic mutations and molecular biomarkers.

Cancer genomics, as defined by the American Cancer Society, examines a person’s entire genetic makeup and its interactions within cellular and environmental contexts. Genomics is fundamental to understanding and treating cancer, as it identifies genetic mutations that drive tumor growth and influence how patients respond to therapy [9,10]. Variations in critical genes can be detected through genetic testing [11,12], allowing for more precise diagnoses and the development of targeted and personalized treatments [10].

The rapidly evolving field of personalized medicine has gained substantial attention worldwide; it is not simply a new technology but a new approach to the practice of medicine. However, personalized medicine and genetic testing rely heavily on patient-specific data, which raises challenges for their effective development and clinical translation, particularly regarding data privacy, regulatory oversight, and healthcare infrastructure requirements.

In Saudi Arabia, personalized medicine is becoming more common, supported by advancements in the healthcare infrastructure. Key initiatives were launched to improve personalized care and position the Kingdom as a global leader in genomics while supporting national strategies for cancer control and health promotion under the umbrella of Vision 2030 and the National Transformation Program [13].

To the best of our knowledge, no comprehensive review has been published to date that specifically addresses the challenges of using genomic data for personalized medicine in oncology in Saudi Arabia. However, we acknowledge that this study is not intended to be comprehensive; rather, we provide an exploratory first assessment based on expert interviews and narrative regulatory mapping to identify preliminary themes and gaps requiring further investigation. Therefore, we aimed to conduct an exploratory examination of the existing regulations, implementation practices, and challenges associated with using genomic data for personalized oncology in Saudi Arabia. Moreover, we offer actionable recommendations, suggest solutions, and outline future directions for advancing the use of genomic data in personalized oncology in Saudi Arabia.

## 2. Materials and Methods

### 2.1. Methodology

This study was conducted in two stages using a mixed-methods exploratory design, not a comprehensive analytical framework. In Stage 1, qualitative structured interviews were conducted with subject matter experts (SMEs) using an interview guide developed based on the existing literature. To complement this methodological approach, a non-systematic literature review was performed to support the study’s exploratory findings. The literature review was conducted using PubMed and Google Scholar, focusing on articles published from 2015 to 2025. Search terms included: “Personalized medicine and Regulations” OR “Personalized medicine and Implementations” OR “Personalized medicine and Challenges” OR “Personalized medicine and Cancer driver genes” OR “Personalized Oncology and Genomic data” OR “Genes and Cancer incidence in Saudi Arabia” OR “Genomic medicine and Cancer” OR “Genomic data and Regulations in Saudi Arabia” OR “Artificial Intelligence (AI) and Cancer in Saudi healthcare” OR “Personal data protection and laws” OR “Health transformations and Cancer.” A total of 34 studies were selected based on relevance to the research objectives; however, formal inclusion/exclusion criteria, PRISMA screening, and quality assessment were not applied, representing a limitation of this review.

Stage 2 focused on a narrative review of publicly available regulatory documents from stringent regulatory authorities, such as the United States Food and Drug Administration (FDA) and European Medicines Agency (EMA), along with relevant stakeholders in Saudi Arabia, including regulators from SFDA, MOH, SDAIA, Saudi National Institute of Health (Saudi NIH), King Abdulaziz City for Science and Technology (KACST), King Abdullah University of Science and Technology (KAUST), King Abdullah International Medical Research Center (KAIMRC), and Saudi Health Council (NCC and National Committee of Bioethics [NCBE]), in addition to healthcare providers from government hospitals, such as King Faisal Specialist Hospital and Research Center (KFSHRC) and National Guard Hospital (NGH).

Regulatory Comparison Framework: Table 1 demonstrates a descriptive listing of regulatory instruments from the FDA, EMA, and Saudi stakeholders. However, we acknowledge that this table does not incorporate a formal analytical framework (e.g., comparative law analysis, policy matrix scoring, or dimensional analysis). The dimensions differ across jurisdictions because regulatory systems evolved independently with different legislative structures. FDA guidance documents are non-binding recommendations, EMA regulations are legally binding directives and regulations, and Saudi instruments include laws, guidelines, and draft policies at various stages of implementation. Future research should be aimed at applying structured comparative frameworks (e.g., World Health organization, WHO regulatory system strengthening metrics or the Organization for Economic Co-operation and Development [OECD] Regulatory Indicators Questionnaire) to enable systematic cross-jurisdictional analysis.

**Table 1:** Comparison of current regulations between the FDA and EMA.

FDA	EMA
Genetic Database Recognition Decision Summary for ClinGen Expert Curated Human Variant Data [14]	Regulation (EC) No 1394/2007 of the European Parliament and of the Council of 13 November 2007 on Advanced Therapy Medicinal Products and Amending Directive 2001/83/EC and Regulation (EC) No 726/2004 [15]
Considerations For Design, Development, and Analytical Validation of Next Generation Sequencing (NGS)-Based In Vitro Diagnostics (IVDs) Intended to Aid in the Diagnosis of Suspected Germline Diseases Guidance for Stakeholders and Food and Drug Administration [16]	Regulation (EU) 2016/679 of the European Parliament and of the Council of 27 April 2016 (General Data Protection Regulation) [17]
Precision Medicine Initiative: Data Security Policy Principles and Framework [18]	Regulation (EU) 2022/868 of the European Parliament and of the Council of 30 May 2022 on European Data Governance and Amending Regulation (EU) 2018/1724 (Data Governance Act) [19]
Guidance for Industry Pharmacogenomic Data Submissions [20]	Regulation (EU) 2023/2854 of the European Parliament and of the Council of 13 December 2023 on Harmonized Rules on Fair Access to and Use of Data and Amending Regulation (EU) 2017/2394 and Directive (EU) 2020/1828 (Data Act) [21]
Oncology Drug Products Used With Certain In Vitro Diagnostic Tests: Pilot Program [22]	Regulation (EU) 2025/327 of the European Parliament and of the Council of 11 February 2025 on the European Health Data Space and Amending Directive 2011/24/EU and Regulation (EU) 2024/2847 [23]
Use of Public Human Genetic Variant Databases to Support Clinical Validity Draft Guidance for Genetic and Genomic-Based In Vitro Diagnostics [24]	Guidelines on Good Clinical Practice Specific to Advanced Therapy Medicinal Products [25]
E18 Genomic Sampling and Management of Genomic Data Guidance for Industry [26]	ICH Guideline E18 on Genomic Sampling and Management of Genomic Data [27]

EMA, European Medicines Agency; FDA, United States Food and Drug Administration; ICH, International Council for Harmonization of Technical Requirements for Registration of Pharmaceuticals for Human Use.

### 2.2. Study Design

This study was an exploratory review conducted using a mixed-methods approach. This method was selected to provide preliminary insights into the research problem by integrating complementary data sources, thereby enriching the findings and facilitating hypothesis generation for future research. We acknowledge that this design does not support generalizable conclusions but rather provides in-depth exploratory findings. Similar study designs have been used previously in policy research and regulatory science to generate preliminary insights before large-scale quantitative studies [28,29].

### 2.3. Qualitative Structured Interview

#### 2.3.1. Data Collection and Interview

The interview comprised several predetermined questions organized into five key themes to identify existing and missing regulations and gather qualitative insights into the regulatory implications of using genomic data in personalized oncology in Saudi Arabia. Additionally, we discussed potential solutions, gathered recommendations for future directions, and validated the findings of the literature review. The interviews took place in February 2025 and were conducted both in person and online using Microsoft Teams and Zoom. Each interview was conducted individually, audio-recorded, and subsequently transcribed and organized according to the key themes.

#### 2.3.2. Participant Selection Criteria

Owing to the scarcity of experts in this domain in Saudi Arabia, five individuals were invited to participate in this study. The participants were nominated based on recommendations from Dr. Khalid Almoaikel (co-author), given his familiarity with multiple experts in the field. We acknowledge that this convenience sampling approach introduces potential selection bias and limits representativeness. Future studies should employ stratified sampling across all stakeholder groups (regulators, clinicians, researchers, ethicists, and industry representatives) and multiple recruitment channels to reduce nomination bias. Four of these five individuals agreed to participate as subject-matter experts (SMEs). The selection was based on at least 10 years of professional experience and aimed to ensure representation from the governmental and private sectors in Saudi Arabia. Although this purposive sampling strategy was appropriate for exploratory qualitative research, we recognize that four participants cannot adequately represent the full diversity of Saudi healthcare providers, regulators, and oncology specialists. Therefore, our findings should be interpreted as preliminary insights from experienced stakeholders rather than as nationally representative conclusions.

The experts had different levels of expertise across various fields, including personalized medicine, medical oncology, data science, bioethics, and pharmaceutical quality assessment.

#### 2.3.3. Data Collection

Each qualitative structured interview lasted approximately 45 min, except for one that lasted 2 h because the expert provided particularly comprehensive information. Each interview was conducted in English using a structured and coherent process. A structured discussion guide developed in alignment with the research objectives served as the foundation for conducting the interviews. Qualitative data were subsequently processed, cleaned, and organized into Microsoft Word files for data analysis.

#### 2.3.4. Themes of the Qualitative Structured Interview

We developed 19 questions focusing on current practices, challenges, barriers, solutions, and future recommendations. The participants were guided by clear themes and predetermined questions while allowing for follow-up inquiries to clarify responses and obtain additional relevant information. The structured interview, comprising five themes and their corresponding key questions, is presented in Table A1 (Appendix A).

#### 2.3.5. Data Extraction and Thematic Analysis

Thematic analysis was conducted using a structured approach to ensure rigor and transparency, and qualitative reporting adheres to the Consolidated Criteria for Reporting Qualitative Research (COREQ) guidelines (see Supplementary Materials, Table S1: COREQ 32-Item Checklist) [30]. The process involved the following steps: (1) Familiarization: All interview transcripts were read repeatedly by the primary researcher (L.H.) to achieve immersion in the data. (2) Initial Coding: A preliminary coding framework was developed based on the interview guide themes (demographics, current practices, challenges, recommendations, and future perspectives). Transcripts were coded line by line using NVivo software (version 14, QSR International), 35 Corporate Drive, Burlington, MA, USA. (3) Theme Development: Codes were collated into potential themes through iterative comparisons across transcripts. Themes were reviewed for internal homogeneity and external heterogeneity. (4) Refinement: Theme names and definitions were refined to accurately capture the essence of each theme. (5) Triangulation: Emerging themes were triangulated with findings from the literature review and regulatory document analysis to enhance credibility. (6) Reporting: Final themes were mapped to the research objectives.

### 2.4. Literature Review

A non-systematic, targeted literature review was conducted using PubMed and Google Scholar, focusing on articles published between 2015 and 2025. In total, 34 studies were selected based on their relevance to the research objectives to support the findings of this review. The selection process involved the following steps: (1) initial searches using the specified terms; (2) screening of titles and abstracts for relevance to personalized oncology, genomics regulation, and the Saudi healthcare context; (3) full-text review of potentially relevant articles; and (4) inclusion of articles providing regulatory, implementation, or challenge-related insights. However, we did not apply formal PRISMA guidelines, systematic screening protocols, or quality assessment tools (e.g., Critical Appraisal Skills Programme [CASP], A Measurement Tool to Assess Systematic Reviews [AMSTAR]). The initial search yielded approximately 150 records; after removing duplicates and screening, 34 articles were retained. Reasons for exclusion were not systematically documented, representing a methodological limitation. Future reviews should employ a systematic review methodology with registered protocols (e.g., PROSPERO registration) and comprehensive quality assessment.

The search strategy utilized the terms detailed in Section 2.1. This method enabled us to understand stakeholder perspectives and uncover the existing regulations, implementations, and challenges of using genomic data in personalized oncology medicine in Saudi Arabia.

### 2.5. Regulatory Review

This study also included a narrative regulatory review of the current guidelines, policies, ethical considerations, and frameworks governing genomic data use and personalized medicine in oncology across key global regulatory agencies, such as the FDA and EMA, and relevant local stakeholders in Saudi Arabia. We aimed to examine existing official documents, published regulations, and draft policies to identify thematic areas rather than to conduct a systematic comparative analysis.

## 3. Results

### 3.1. Expert Opinions

The findings of this review integrate qualitative insights from SMEs with supporting evidence from the non-systematic literature review, narrative published documents from relevant stakeholders in Saudi Arabia, and reference to stringent FDA and EMA regulations. To ensure a comprehensive exploratory analysis, expert opinions were triangulated using published papers and regulatory documents.

### 3.2. Thematic Analysis Overview

The findings from SMEs and the literature review, along with the regulatory overview, are presented in an aggregated manner, encompassing the following six primary themes: (1) Demographics and Background, (2) Current Practices and Implementation, (3) Challenges and Barriers, (4) Recommendations and Solutions, (5) Future Perspectives, and (6) Regulatory Comparison. Additional sub-themes were identified within these primary categories.

### 3.3. Demographics and Background

Four SMEs participated in this study. Two experts represented the governmental sector, whereas the remaining two had experience in both governmental and private sectors. Table 2 presents the demographic characteristics of the participating experts.

### 3.4. Current Practices and Implementation

Historically, cancer treatment followed a one-size-fits-all approach, often referred to as “cut, burn, and poison.” These treatments may affect both cancerous and healthy cells, causing considerable adverse effects. Additionally, in some cases, cancers develop resistance to chemotherapy, and in other situations, non-disease-related mortality is observed [31]. Personalized medicine has emerged as a transformative approach for tailoring treatments based on patient- and disease-specific factors. This approach relies on genetic testing and molecular profiling to identify specific mutations, thereby enabling the use of targeted therapies and immunotherapies.

Accordingly, experts were asked to share their perspectives on the application of personalized medicine in cancer treatment. All experts agreed that personalized medicine is the future of healthcare, a life-saving advancement, and the ultimate goal of cancer treatment. This is consistent with published research indicating that numerous researchers in the oncology community support the adoption of personalized medicine [5]; however, we note that this consensus among four experts does not represent the full spectrum of Saudi oncology practice.

The Saudi Press Agency reported that, in alignment with national medical innovation strategies, the Kingdom is leveraging AI technologies to enhance healthcare and quality of life, with an investment of millions of dollars to accelerate healthspan science research. This strategy aims to achieve 15 years of progress in just 3 years, reflecting Saudi Arabia’s commitment to healthcare leadership and innovation [13].

#### 3.4.1. Healthcare Providers

**Figure 1:**
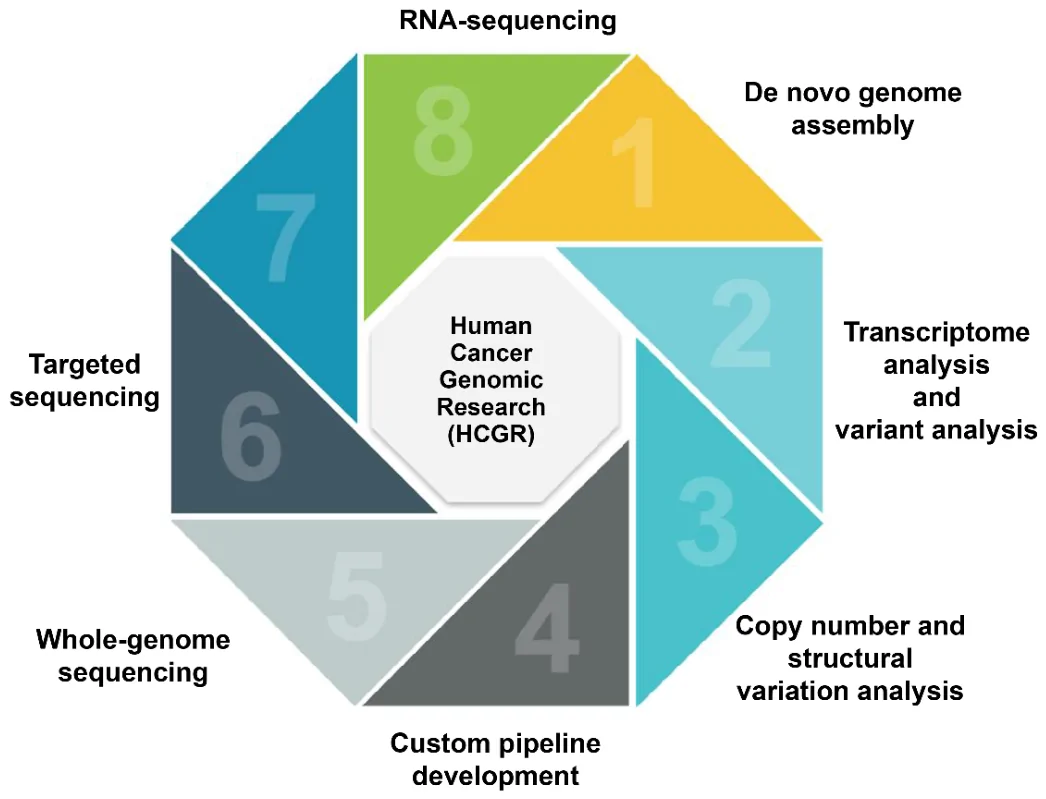
Human Cancer Genomic Research (HCGR) laboratory at King Faisal Specialist Hospital & Research Centre. This laboratory specializes in identifying various genes and signaling pathways involved in cancer and offers a range of bioinformatics and high-throughput sequencing services, including: (1) de novo genome assembly, (2) transcriptome analysis and variant analysis, (3) copy number and structural variation analysis, (4) custom pipeline development, (5) whole-genome sequencing, (6) targeted sequencing, (7) RNA-sequencing, and (8) additional specialized analyses.

In alignment with Vision 2030, precision medicine is considered a key goal, and multiple initiatives have been created to improve healthcare practices in Saudi Arabia [13]. For instance, the experts stated that a dedicated center has been established for advanced genetic treatments to enable cell extraction, genetic modification, and reinfusion into patients. Major hospitals such as the NGH and KFSHRC are leading efforts in genetic testing. These hospitals have established in-house capabilities, following specific protocols for genetic testing and treatment decisions, allowing for faster turnaround and reducing the waiting time for genetic test results from 3 weeks to 7–10 days (Figure 1). This improvement enables timely treatment adjustments and improves patient outcomes. Additionally, the training of healthcare providers varies depending on hospital-specific programs and individual experiences with pharmacogenomic testing.

#### 3.4.2. Patients

The experts highlighted that Saudi Arabia has one of the strongest informed consent policies, largely because of the individualistic approach embedded in this ethical framework, which effectively ensures that individual autonomy is respected in genetic and healthcare research. Large hospitals such as the NGH and KFSHRC employ health educators who play a vital role in patient education and explain treatment plans, potential risks, and expected outcomes to patients and their families, thereby fostering informed decision-making.

Quantitative Context: While experts reported strong informed consent policies, we acknowledge that no quantitative data (e.g., patient comprehension surveys, consent form readability scores, or institutional audit data) were collected to validate these claims. Future research should incorporate patient-reported outcome measures and health literacy assessments to strengthen these findings.

#### 3.4.3. Collaboration Among Stakeholders

Collaborations between experts in different fields [32], as well as between local entities and researchers from research-intensive nations, are essential for the success of population-wide genome projects [33]. Therefore, experts have emphasized that collaboration in genomic research and personalized medicine remains limited in Saudi Arabia, primarily within select institutions. Research entities such as the KAIMRC have collaborated with the pharmaceutical industry in areas such as genomic and kinetic-genetic data. However, the experts acknowledged that nationwide collaboration can only be achieved once comprehensive regulations are in place.

Quantitative Context: The assessment of collaboration limitations is based on expert perceptions rather than on bibliometric analysis, network mapping, or institutional partnership surveys. Systematic analysis of co-authorship networks, funding collaboration patterns, and data-sharing agreements would provide more robust evidence for this finding.

#### 3.4.4. Regulations, Ethics, and Data Privacy

In the context of data privacy, genetic privacy has been recognized as both an ethical and legal principle that safeguards personal data [11]. Genetic information is highly sensitive and may lead to social and economic consequences. This was stated by expert SME-03, who recognized that privacy and data handling in Saudi Arabia are governed by the Personal Data Protection Law (PDPL) issued by SDAIA, and genomic data are classified as personal data. Moreover, a general bioethics policy exists at KACST. In the regulatory landscape of Saudi Arabia, expert SME-04 asserted that the practice currently follows Good Manufacturing Practice and Chemistry, Manufacturing, and Control guidelines for advanced therapies.

The experts acknowledged that treatment guidelines for cancer and personalized medicine exist. Nonetheless, the use of genomic data in oncology practice in Saudi Arabia is uncommon, and experts have pointed out that the main reason is the lack of a comprehensive regulatory framework governing the use of data, particularly for secondary utilization. Additionally, they emphasized that, while efforts have been made, the regulatory framework for genomic data remains underdeveloped, and existing regulations require refinement to ensure their effectiveness in addressing genomic data and personalized medicine applications.

#### 3.4.5. Success and Failure Stories Regarding the Role of Genomic Data in Cancer Care to Advance Personalized Medicine

Advancements in personalized medicine have led to significant success stories, one of which is chimeric antigen receptor (CAR)-T cell therapy. This treatment has been a breakthrough in treating certain types of cancers (e.g., leukemia and lymphoma) by genetically modifying a patient’s T cells to target cancer cells. Similarly, genomics has been applied in other areas such as depression treatment, where genetic profiling helps identify the most effective antidepressants to improve patient outcomes. However, because of the complexity of cancer, challenges persist, as seen in the failures of CAR-T-cell therapy, notably in the Rocket trial, in which unexpected adverse reactions resulted in the death of five patients with acute lymphoblastic leukemia.

These findings are consistent with those published by the National Cancer Institute, highlighting that CAR-T-cell therapy has shown remarkable effectiveness in blood cancers, particularly in children with relapsed acute lymphoblastic leukemia and adults with non-Hodgkin lymphoma and multiple myeloma. Clinical trials have reported long-term survival and cure for some patients. CAR-T-cell therapy can lead to adverse effects, such as cytokine release syndrome, which can be life-threatening in rare cases. In most children and adults, cytokine release syndrome can be controlled with tocilizumab (Actemra) and, if required, steroid therapy [34].

#### 3.4.6. Initiatives in Genomic Data and Personalized Medicine for Oncology in Saudi Arabia, as Outlined by Experts

In November 2023, the SDAIA, along with a global healthcare consultancy firm, led an initiative to establish a comprehensive legal framework for genomic data management in Saudi Arabia. The project aimed to develop a long-term vision for genetic data governance, assess existing regulations, identify policy gaps by benchmarking against global standards, and explore the feasibility of a centralized national bioinformatics center. The methodology involved reviewing Saudi genomic regulations, conducting comparative analyses with leading countries (the United Kingdom, the European Union, the US, and China), and providing recommendations for strengthening Saudi Arabia’s genomic policies. The outcome of this initiative is a comprehensive report outlining regulatory gaps and strategic recommendations, including insights into the establishment of a national bioinformatics center. This initiative remains underdeveloped and is being discussed by higher authorities.

Similarly, the NCBE at the Saudi Health Council has taken significant steps to regulate the ethical and legal use of secondary data by establishing a dedicated committee and adopting the Findable, Accessible, Interoperable, Reusable (FAIR) principles to enhance data transparency and usability for researchers. As part of this initiative, efforts have been made to develop a trusted data hub to facilitate data sharing and support research, as well as to organize an expert workshop to address regulatory and ethical issues in personalized medicine. However, this project has encountered challenges and remains under development.

Another initiative of the Saudi Health Council is Nahar Saab, a platform developed for data storage and dispensing. It is a successful model that operates at the policymaker level, rather than being designed for individual patient-level data management.

The SFDA has created three draft guidelines that are being prepared for public consultation alongside a clinical team working on guidelines addressing patient data privacy and long-term use of advanced therapies.

### 3.5. Challenges and Barriers

#### 3.5.1. Utilizing Genomic Data and Personalized Medicine in Oncology: Healthcare Providers, Patients, and Stakeholder Collaboration

The experts have noted that personalized medicine exists at the border between research and clinical practice. It is not purely a medical practice, as it involves genetic analysis and customized healthcare planning, nor is it purely research, as its ultimate goal is direct patient treatment. This hybrid nature leads to uncertainty in defining the regulatory authority. According to the experts, the real issues revolve around cost and data protection for individuals. High costs and logistical complexities present significant barriers to widespread implementation. Extensive genomic testing places a financial burden on hospitals, with costs encompassing laboratory readiness, test kits, and drug availability, rendering treatment ineffective if the required drugs are not available.

Quantitative Context: Cost-related barriers are reported based on expert estimates rather than on health economic analyses, institutional budget data, or patient expenditure surveys. Formal health technology assessment (HTA) and cost-effectiveness analyses are needed to accurately quantify these barriers.

#### 3.5.2. Healthcare Providers

The experts identified key challenges faced by healthcare providers in implementing personalized oncology medicine. The rapid evolution of oncology care makes it difficult for physicians to stay updated, and pharmacists and physicians lack the formal competency requirements for genetic testing and targeted therapies. Training deficiencies among healthcare providers, delayed test results owing to infrastructure gaps that force some hospitals to send samples abroad, and restricted access to targeted therapies create additional barriers that lead to treatment delays. This aligns with published studies showing that limited digitalization of data and inadequate training of healthcare professionals hinder the effective implementation of personalized medicine in developing healthcare systems.

Quantitative Context: Training deficiencies and infrastructure gaps are identified through expert interviews rather than through competency assessments, skills surveys, or infrastructure audits. Standardized competency frameworks (e.g., the European Society of Medical Oncology’s precision medicine curriculum) and digital maturity assessments are warranted to provide more robust evidence.

The experts have illustrated that frequent updates to oncology guidelines require continuous education, whereas staffing shortages in genetic and laboratory testing limit the effective implementation of personalized medicine. Tracking genetic mutations and matching them with treatments are complex tasks. Although artificial intelligence (AI)-assisted tools for mutation-to-drug matching are gaining global traction, their local adoption remains limited. Addressing these challenges requires investments in technology, education, and infrastructure to enhance personalized oncology care in Saudi Arabia.

#### 3.5.3. Patients

The experts outlined various obstacles that patients encounter in personalized medicine, particularly in understanding genetic data and their implications. The experts agreed that many patients are unaware of the sensitivity of their genetic information and have only a basic understanding of genetic testing, with minimal involvement in treatment decisions. Additionally, not all patients receive detailed explanations regarding their treatment, and hospital resources influence the level of patient education. Insurance- and cost-related barriers further complicate access to care, with some treatments being rejected without valid justification.

Another major challenge is the lack of up-to-date, culturally appropriate educational materials in Arabic. The absence of standardized patient education processes, especially in smaller hospitals and remote areas, contributes to this issue, hindering patient comprehension and informed decision-making. Bridging these gaps is crucial to enhancing patient awareness and facilitating engagement in personalized oncology care.

Quantitative Context: Patient awareness and education gaps are reported based on expert clinical observations rather than on patient surveys, health literacy assessments, or educational material evaluations. Structured patient-reported experience measures and health literacy instruments (e.g., REALM—Rapid Estimate of Adult Literacy in Medicine, NVS—Newest Vital Sig) would strengthen these findings.

#### 3.5.4. Collaboration Among Stakeholders

The experts also emphasized multiple challenges in fostering collaboration among stakeholders in personalized oncology care in Saudi Arabia. In addition to data accessibility issues, many researchers and institutions are hesitant to share data and treat them as proprietary assets, which restricts their availability and slows the progress of collaborative research. Hospitals also impose strict controls on patient data, even when anonymized, making it difficult for external entities, such as AI developers, to utilize this information to advance personalized treatments. The absence of clear regulations and a national framework for data sharing further complicates cooperation among hospitals, businesses, and research organizations. Another issue is limited effective collaboration among healthcare providers across different institutions.

In summary, the absence of a clear regulatory framework limits broader collaboration among stakeholders in Saudi Arabia. The experts believe that establishing regulations will drive infrastructure development, creating a more integrated ecosystem for personalized medicine that enhances both the healthcare system and stakeholder engagement.

Quantitative Context: Collaboration barriers are assessed through expert perceptions rather than through network analysis, data-sharing agreement audits, or bibliometric studies. Social network analysis and institutional collaboration mapping are warranted to provide more objective evidence.

#### 3.5.5. Regulations, Ethics, and Data Privacy

Despite advancements in personalized medicine, experts have drawn attention to the lack of a national regulatory framework in Saudi Arabia, which has led to fragmented policies and inconsistent data governance. The absence of standardized guidelines for data storage and secondary use hinders progress in healthcare, research, and business areas. Challenges in balancing regulations, particularly those governing direct-to-consumer genetic testing, have raised privacy concerns. Ethical issues, such as data commodification and individual autonomy versus national data ownership, further complicate the situation. Trust in institutions that handle sensitive health data is crucial for preventing privacy breaches and ensuring ethical data utilization.

Expert SME-04 noted that the existing regulations for Advanced Therapy Medicinal Products (ATMPs) largely depend on EMA guidelines owing to the absence of locally developed policies. Given the complexity of ATMPs, specialized knowledge and a unified international framework for advanced therapies are essential for regulators to establish clear guidelines. As a member of the International Council for Harmonisation of Technical Requirements for Registration of Pharmaceuticals for Human Use (ICH), the SFDA is taking steps toward global leadership in regulatory harmonization, aiming to bridge existing gaps in international ATMP regulation.

Many studies have reported uncertainty regarding privacy awareness [28] and genetic data security [29], emphasizing the need for standardized data collection, regulated sharing, and ethical considerations to support AI-driven disease diagnosis and treatment optimization.

#### 3.5.6. Effects of the National Regulatory Requirements on the Use of Genomic Data

The experts indicated the need for a standardized regulatory framework for genomic data use in Saudi Arabia’s healthcare system. The NPHIES initiative of the MOH plays a key role in facilitating data exchange; however, national guidelines for genetic testing in cancer care lack consistent implementation. The establishment of in-country genomic testing capabilities and regulatory frameworks is essential for reducing treatment delays and improving patient outcomes.

The role of the SFDA should include mandating that drug labels specify the required genetic tests for personalized medicine and aligning regulations with the testing requirements to support clinicians in prescribing targeted therapies. Clinical trials overseen by the SFDA must comply with bioethical regulations. However, no comprehensive consent framework for genomic testing currently exists. In addition, the SFDA classifies genetic and genomic tests as medical devices, reinforcing the need for clear regulations governing their use in diagnostics and treatment planning. Ultimately, further regulatory improvements are needed, including better consent forms, national data management policies, and streamlined approval processes for new medications, to enhance efficiency and reduce delays in patient access to personalized treatments.

### 3.6. Recommendations and Solutions

The experts emphasized the crucial role of genomic data in personalized oncology, highlighting their importance in modern healthcare. Cancer is not a single disease but a collection of diverse malignancies, each with unique genetic mutations and microenvironments, making standardized treatments unsuitable for all patients. Expert SME-02 noted that cancer evolves over time and often adapts to resist treatment, which underscores the need for personalized approaches. Genomic data help select the most effective treatments, predict toxicities, and tailor dosages; these applications are central to pharmacogenomics. As genetic testing becomes increasingly vital, integrating genomic data into cancer care is essential for precision medicine and ultimately for improving patient survival and quality of life.

Experts emphasized the need for a structured national regulatory framework to oversee personalized medicine, with a dedicated regulatory body that ensures consistency and clarity in genomic data governance. The SDAIA has initiated efforts to regulate genomic and healthcare data; however, the experts stressed the necessity for a central authority to bridge regulatory gaps. Establishing clear guidelines that balance innovation, patient rights, and ethical considerations is essential for defining data ownership responsibilities. The experts also highlighted the importance of a national data-sharing platform for the effective implementation of personalized medicine. Transparency, the protection of patient privacy, and ethical data use remain key priorities in fostering trust and maximizing the benefits of genomic data in personalized oncology.

To enhance implementation, the experts recommend increasing education and awareness, establishing national registries to track cancer prevalence, and investing in structured training programs for healthcare professionals. Streamlining the laboratory infrastructure and drug procurement would improve test availability and reduce treatment delays. Addressing the high costs of personalized medicine through specialized hospital hubs, HTAs, and outcome-based agreements with pharmaceutical companies might make treatment more accessible. Equally important, the experts called for AI integration to support clinical decision-making and the creation of a national tumor board to standardize treatment approaches. A unified system under the MOH with centralized resource sharing and regulatory oversight would significantly optimize personalized medicine across Saudi Arabia.

Limitation Note: These recommendations are derived from expert opinions and a narrative regulatory review rather than from evidence synthesis from systematic reviews, cost-effectiveness analyses, or implementation science frameworks. Although informed by extensive experience, their feasibility and impact require validation through pilot studies, stakeholder consultations, and health economic modeling.

Expert SME-04 expressed the need for continuous improvement of the ATMP regulatory framework, including the Good Tissue Practice and Good Cell Practice guidelines. Strengthening the regulatory environment requires an interdisciplinary approach to align with international standards, including the establishment of an ATMP committee that involves multidisciplinary experts. This approach will foster collaboration among researchers, manufacturers, and clinicians, ensuring patient safety and sound regulatory decisions. Training programs on new production methods are necessary to address the expertise gap in Saudi Arabia. The SFDA’s involvement in global regulatory bodies, such as the ICH, will enhance its leadership in ATMP regulation. The ATMP committee’s recent developments in personalized medicine guidelines further emphasize the need for Saudi Arabia to establish its own ATMP regulatory structure to ensure product quality, patient safety, and effective oversight.

Genomic medicine is an interdisciplinary specialty involving the use of a person’s genomic information. To position Saudi Arabia as a regional leader in genomic medicine, expert SME-01 underscored the importance of collaboration between the pharmaceutical industry and healthcare sectors to ensure that access to patient data is used for advancing medical research while protecting privacy and preventing financial exploitation. In addition, the need for two levels of access was highlighted: individual-level access for specific patient care, ensuring minimal data exposure; and public-level access to genomic databases, which requires stricter regulations to prevent misuse. Ethical considerations were stressed, including the co-ownership of data to secure fair benefits for Saudi Arabia, safeguards against pharmaceutical companies reselling treatments derived from local data at high costs, and ensuring alignment with national health objectives and economic interests.

Other experts have confirmed the role of the SFDA in accelerating drug approvals for personalized medicine, especially for urgent oncology treatments. Enabling localized research on drug efficacy and increasing bioinformatics research is deemed essential for evaluating drug responses in the Saudi population because many treatments are currently being tested in Western populations, which may not reflect Saudi genetic diversity and drug metabolism profiles.

### 3.7. Future Perspectives

The experts envisioned a future in which every patient with cancer in Saudi Arabia will undergo comprehensive genetic profiling to enable truly personalized treatments. The experts acknowledged that personalized medicine is the future, necessitating well-regulated DNA and tissue banks. Expert SME-02 stressed the role of entrepreneurs and businesses in developing decision support tools for clinicians, noting that successful models from other regions could be adapted to Saudi Arabia. Although the country’s healthcare startup ecosystem is still developing, the potential for growth and innovation in this space is strong. Additionally, pharmacogenomics has been identified as a crucial tool for determining patient responsiveness to specific drugs, further enhancing personalized oncological care.

Expert SME-04 shared insights into the future of personalized medicine and the role of the SFDA in advancing this field. The integration of research data from centers across the Kingdom is essential for the development of personalized medicine, particularly for cancer treatment. By consolidating these data, customized therapies can be developed for various types of cancer. This is part of the SFDA Vision Strategy, aiming to integrate all research data generated from Saudi Genome Center and other research centers to develop customized or individualized personalized medicine for different types of cancer in the future. The SFDA is actively working on horizon-scanning to predict upcoming trends and ensure preparedness. Overall, the future of genomics in Saudi Arabia holds great promise, and although challenges in regulation, infrastructure, and accessibility persist, they are being progressively addressed through national initiatives and strategic planning.

The experts have presented numerous emerging advancements that will shape the future of genomics and personalized oncology medicine. AI is seen as an inevitable and crucial tool in cancer care, particularly given the complexity of the disease, and will assist in analyzing vast amounts of data and supporting clinicians in managing complex cases. The integration of genomics with other omics, such as metabolomics and proteomics, is expected to revolutionize treatment strategies, particularly for chronic diseases. Oncology vaccines are another hot topic, with companies developing vaccines to prevent cancer, and cancer vaccines being researched for both prophylactic purposes and to alleviate symptoms. Experts have also highlighted the potential of 3D bioprinting in advancing cancer treatment. These innovations, coupled with genomics and precision medicine, hold great promise for the future of oncology in Saudi Arabia, contingent upon the establishment of supportive regulatory frameworks, infrastructure investment, and sustained stakeholder collaboration.

### 3.8. Comparative Analysis of Regulatory Approaches (Descriptive Mapping)

Methodological Note: This section presents a descriptive narrative mapping of regulatory instruments rather than a systematic comparative analysis. We acknowledge that Table 1 demonstrates regulations without applying a formal analytical framework (e.g., comparative law dimensions, policy scoring, or regulatory system metrics). The FDA, EMA, and Saudi regulatory systems differ fundamentally in their legal structures: FDA issues non-binding guidance documents within a federal administrative framework; EMA operates through legally binding EU regulations and directives; whereas Saudi stakeholders operate under a centralized monarchy with emerging regulatory authorities. These structural differences preclude direct one-to-one comparison without a standardized analytical framework, which was beyond the scope of this exploratory study. Future research should be aimed at applying structured comparative methodologies (e.g., the WHO Global Benchmarking Tool for evaluation of national regulatory systems or the OECD Regulatory Policy Outlook framework) to enable meaningful cross-jurisdictional analysis.

Advances in the field of personalized medicine have led to the approval of targeted therapies by global regulators, with molecular testing guiding cancer treatment to improve survival and minimize adverse effects. This section descriptively compares the regulatory instruments of stringent regulatory agencies in their approach to personalized medicine in general, with a specific focus on precision oncology.

The FDA has worked on personalized medicine to pave the way and guide future activities in this field. In 2013, the FDA published “FDA’s Role in a New Era of Medical Product Development” [35], and between 1 August 2017 and 31 July 2018, the FDA approved 14 new anticancer therapeutics and expanded the use of 11 previously approved anticancer treatments to include additional cancer types [34]. Moreover, the FDA launched in January 2015 the Precision Medicine Initiative, which aims to optimize treatment selection for individual patients [36]. Cancer profile testing is important for personalized treatment decisions. Therefore, the FDA announced marketing authorization for three tumor-profiling next-generation sequencing tests alongside the Integrated Mutation Profiling of Actionable Cancer Targets tumor-profiling test, an in vitro diagnostic test. These tests help detect mutations in 468 genes and identify more cancer biomarkers than any previously reviewed tests [37,38]. The FDA views personalized medicine as a way to boost patient benefits while reducing risks by enhancing the safety and effectiveness of medical products [35], which is why multiple guidelines have been issued regarding personalized medicine for cancer treatment. Table 2 demonstrates the guidelines issued by the FDA and EMA related to the use of genomic data and personalized medicine.

The FDA instruments listed are primarily non-binding guidance documents and draft guidance issued under the Federal Food, Drug, and Cosmetic Act. The EMA instruments include legally binding EU regulations and directives adopted through the ordinary legislative procedure. The Saudi instruments (Table 3) include laws, standards, and draft guidelines at various stages of development. These differing regulatory dimensions (binding vs. non-binding, federal vs. supranational vs. centralized) were not systematically analyzed in this exploratory study.

The EMA plays a key role in regulating and enabling anticancer drug development and has approved numerous antineoplastic agents that are now essential in oncology. With innovations in personalized medicine, immunotherapies, and other advanced therapies, oncology has evolved rapidly, offering new treatment options for various cancers. One goal of the EMA is to support treatment optimization research and address the evidence gaps faced by oncologists [39]. Moreover, the International Consortium of Personalized Medicine was launched to effectively implement personalized medicine. In this regard, the EMA initiated the publication of several guidelines on utilizing data and personalized medicine (Table 2).

Saudi Arabia is actively promoting genomics and personalized medicine through various initiatives aimed at improving the citizens’ quality of life and enhancing the nation’s global reputation. The NCC of the Saudi Health Council is considered one of the first national register programs in the Kingdom. It is responsible for collecting, analyzing, and managing cancer data in Saudi Arabia and publishes an annual Cancer Registry report. The center focuses on cancer control, treatment, and healthcare improvement through national initiatives and collaboration across various healthcare sectors and research institutions.

As part of the Ministry of National Guard Health Affairs, the KAIMRC is advancing efforts to join the global hub for personalized medicine to develop and contribute to personalized clinical care in the era of precision medicine. In 2014, a research-based laboratory was established to study genetic and developmental abnormalities. Moreover, the KAIMRC developed the Saudi Health Data Repository, which serves as a national dataset facilitating data sharing and collaboration among researchers by providing access to accurate, updated health-related data across ten domains, one of them being genomics and precision medicine.

**Figure 2:**
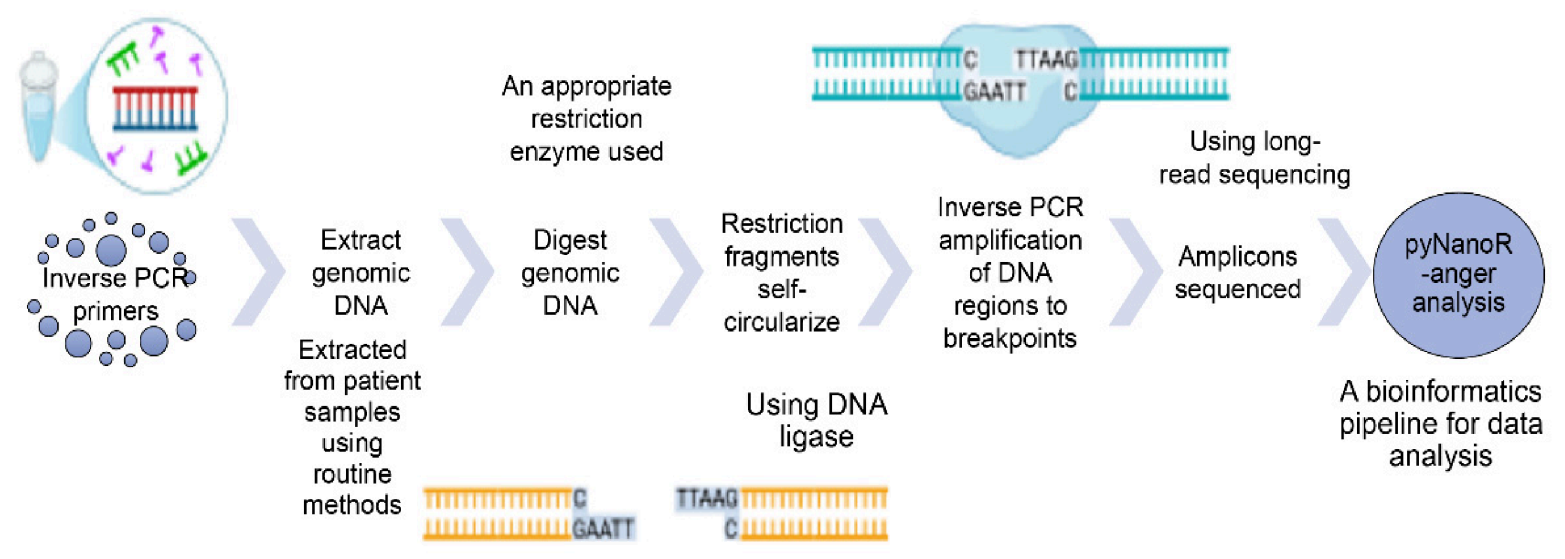
Schematic representation of the NanoRanger technique, which is a nanopore-based technique for the rapid acquisition of neighboring genomic regions. The workflow includes: (1) inverse PCR primers, (2) extract genomic DNA from patient samples using routine methods, (3) digest genomic DNA with an appropriate restriction enzyme, (4) restriction fragments self-circularize using DNA ligase, (5) inverse PCR amplification of DNA regions to breakpoints, (6) amplicons sequenced using long-read sequencing, and (7) pyNanoRanger analysis—a bioinformatics pipeline for data analysis. PCR, polymerase chain reaction. The picture was created using BioRender.com.

The KFSHRC is advancing precision medicine in Saudi Arabia through initiatives to develop protocols, such as a genetic diagnosis protocol that helps CAR-T cell production, a metagenomic sequencing protocol, and a clinical pharmacogenomics service that tailors drug treatments to patients’ genetic profiles. These efforts aim to enhance treatment precision, minimize adverse reactions, and build a comprehensive genomic database for improved patient care. Furthermore, a collaboration has been initiated between KAUST and KFSHRC to develop a new gene sequencing system called NanoRanger (Figure 2). This approach is promising as a cheaper and faster tool for patients with genetic diseases caused by unknown mutations [39,40].

The MOH has a crucial role in activating AI and calls individuals and companies to participate in the “regulatory healthcare sandbox innovation” to accelerate healthcare transformation that aligns with Saudi Vision 2030. Complementary to this, the KACST, in partnership with the NCBE, SDAIA, and SFDA, plays a vital role in developing guidelines and policies for data utilization and personalized medicine, as shown in Table 3.

**Table 3:** Current regulations of relevant stakeholders in Saudi Arabia.

SFDA	SDAIA	KACST & NCBE
Guideline for Good Clinical Practice (GCP) [41]	Data Management and Personal Data Protection Standards [42]	The Regulations of Central Data Bank for Genomic Data [43]
Guide to Good Manufacturing Practice (GMP) for Medicinal Products [44]	Data and Privacy Regulatory Sandbox Guidelines [27]	The Saudi Law of Ethics of Research on Living Creatures and its Implementing Regulations [45]
Guidance on Submission of Chemistry, Manufacturing, and Control (CMC) Information for Cell-based Clinical Trial Applications [46]	The Implementing Regulation of the Personal Data Protection Law [47]	
Regulatory Framework for Drug Approval [48]	Regulation on Personal Data Transfer outside the Kingdom [47]	
Guidelines for Investigational New Drugs (IND) Requirements [49]		
SFDA Guideline on Classification of Advanced Therapy Medicinal Products [50]		

KACST, King Abdulaziz City for Science and Technology; NCBE, National Committee of Bioethics; SDAIA, Saudi Data and Artificial Intelligence Authority; SFDA, Saudi Food and Drug Authority.

The instruments listed represent a mix of finalized guidelines (GCP, GMP), draft guidelines (ATMP classification), and regulatory frameworks under development. The SFDA’s regulatory framework for personalized medicine is evolving, with several draft guidelines pending public consultation. This descriptive listing does not assess implementation status, enforcement mechanisms, or regulatory maturity, which would require a dedicated regulatory system evaluation framework.

## 4. Discussion

Personalized medicine is a promising tool for patients with cancer [46], and genomic data are essential for advancing precision medicine. However, the participating experts identified multiple complexities that hinder the implementation of genomic data and personalized oncology. Therefore, addressing and overcoming these challenges are crucial for the successful implementation of advanced personalized medicine in oncology. The experts outlined key regulatory challenges, noting that despite the crucial role of regulatory bodies in shaping advanced therapy regulations in Saudi Arabia, significant gaps remain. The lack of a national framework, insufficient standardized guidelines for data storage and reuse, logistical complexities, privacy concerns, data protection issues, and lack of data-sharing guidelines all create barriers to the implementation of genomic data and personalized medicine in the Saudi context.

Reliance on EMA regulations is attributed to the absence of locally developed policies. This underscores the need to establish a national regulatory framework and unified international guidelines, develop clear guidelines for ethical considerations, and support the effective implementation of personalized medicine. Our results showed that investing in infrastructure development, ensuring the local availability of genomic testing, and improving access to data and medications could minimize treatment delays. Furthermore, limited collaboration among stakeholders in Saudi Arabia, including healthcare providers, restricts access to genomic data. Collaboration enhances the sharing of genetic data and enables researchers and healthcare providers to improve and accelerate the development of personalized medicine [28]. The results of this study also emphasize the need for stronger cooperation among the pharmaceutical industry, regulatory authorities, and healthcare providers while maintaining data privacy and security standards.

Medical advancements need to be balanced with the protection and privacy of individual data. The results of a previous study are consistent with our results, showing that awareness of the benefits and limitations of using genomic data and the increased training of healthcare providers regarding new advanced medicines will contribute to maximizing the potential of genomic data using personalized medicine [13]. Although attempts are being made to implement personalized treatment strategies, limitations, such as costs and insurance, impose a financial burden and further complicate adoption. The experts have found that directing investments toward personalized medicine can lower healthcare costs by centralizing resources into specialized hubs and adopting approaches, such as HTA, and outcome-based agreements to enhance the affordability of advanced therapies. However, we did not analyze the economic impact of personalized medicine, and health economic analyses are needed to quantify cost-effectiveness and budget impact in the Saudi context.

The experts envisioned a transformative future for oncology in Saudi Arabia, driven by advancements in genomics and personalized medicine. Central to this vision is the establishment of well-regulated DNA and tissue banks, which will serve as vital infrastructure for research and clinical applications, specifically in the oncology field. The integration of genomics with other omics sciences is expected to significantly enhance treatment precision and outcomes [47]. Educational reforms, such as incorporating pharmacogenomics specialists into medical schools, are also underway to build local expertise. In parallel, the integration of AI as a decision support tool is considered fundamental to the future of individualized cancer care. Moreover, 3D bioprinting and personalized cancer vaccines offer promising new treatment modalities [48,49]. These innovations, supported by a growing healthcare startup ecosystem, collectively signal a strategic shift toward a more individualized technology-driven approach to oncology in the Kingdom of Saudi Arabia. We believe that integrating genomic data and personalized medicine into oncology care will enable a future in which each patient is treated as a unique case, with a genetic profile tested across a wide range of genes, and treatments are personalized to their specific molecular and clinical characteristics.

Future Research Directions: In this exploratory study, we identify several priorities for future research: (1) large-scale quantitative surveys of healthcare providers, patients, and regulators to validate the challenges identified; (2) systematic reviews and meta-analyses of personalized oncology implementation in the Middle East; (3) health economic evaluation of genomic testing and targeted therapies in the Saudi population; (4) bibliometric and network analysis of research collaboration patterns; (5) patient health literacy and educational needs assessment; (6) regulatory system benchmarking using international frameworks (WHO, OECD); (7) pilot implementation studies of pharmacogenomic testing in Saudi oncology centers.

This study has some limitations that should be considered in future research. The number of experts involved was limited (n = 4), and participant selection was based on their background, professional experience, and prior knowledge, rather than on standardized selection framework, which introduces potential nomination bias. Additionally, no systematic literature review has been conducted, and the literature review was not reported according to PRISMA guidelines. Furthermore, the comparative regulatory analysis focused primarily on the FDA, EMA, and selected stakeholders in Saudi Arabia, which may limit the generalizability of the findings to other regulatory environments. Moreover, the regulatory comparison was descriptive rather than analytical. These limitations were primarily due to time constraints. Furthermore, the results are closely tied to the specific contexts and experiences of the participants and may be influenced by subjective interpretation or potential gaps in their knowledge of certain regulatory domains.

Additionally, we acknowledge the following limitations in our analytical approach: (a) coding was conducted by a single researcher, which may introduce coder bias—inter-rater reliability was not assessed; (b) no member-checking was conducted with participants to validate theme interpretation; (c) the software-assisted coding was supplemented with manual review to ensure contextual accuracy. Future studies should be aimed at employing multiple coders, establishing inter-rater reliability (e.g., Cohen’s kappa > 0.8), and conducting member-checking to strengthen analytical rigor.

Moreover, this regulatory review was limited to publicly available documents and may not reflect internal policy discussions or draft regulations not yet published; the study did not include patient perspectives, limiting understanding of patient-reported barriers; conclusions about national-level challenges are inferred from expert opinions rather than validated through quantitative surveys, institutional audits, or national health data; and no industry stakeholders were interviewed, potentially missing commercial perspectives on market access and drug availability.

## 5. Conclusions

We used a mixed-methods exploratory approach combining expert interviews with a narrative regulatory review to examine the current regulatory landscape, practical implementation, and challenges associated with the use of genomic data in personalized oncology in Saudi Arabia. Through a non-systematic literature review, expert interviews, and analysis of existing regulations, six key thematic areas were identified, offering a preliminary understanding of both barriers and potential solutions. Although significant challenges persist, the participating experts proposed recommendations to advance the integration of genomic data and personalized medicine into cancer care. We highlight that the effective implementation of personalized medicine requires robust infrastructure, refinement of existing policies, development of clear national guidelines, and the establishment of a central regulatory authority to oversee personalized medicine. Equally important are educational initiatives and multi-stakeholder collaboration, with a strong emphasis on data privacy. Although these findings are based on a limited expert sample and require validation through broader quantitative studies, they provide a foundational roadmap for future research and policy development. Collectively, these findings may guide future efforts to advance precision oncology in the Kingdom of Saudi Arabia, provided that the identified methodological limitations are addressed and the preliminary findings are validated and expanded through further research.

## Figures and Tables

**Table 2:** Demographic characteristics of participating in SMEs.

Expert ID	Area of Specialization	Years of Experience	Sex	Education Level	Sector	Role in Genomic Policy/Oncology Implementation
SME-01	Bioethics & compliance	+25	Men	PhD in Biomedical Science PhD in Bioethics	Government	Involved in bioethics and compliance oversight, including engagement with institutional review processes and ethical guideline development relevant to genomic research.
SME-02	Healthcare provider specialized in hematology/oncology & stem cell transplantation, as well as in pharmacogenomics.	+10	Women	Bachelor’s degree in Pharmaceutical Sciences PharmD from the University of Illinois Chicago Two-year residency in hematology/oncology in Boston	Private	Directly involved in the clinical implementation of pharmacogenomics and hematology/oncology care, applying genomic data at the point of patient treatment.
SME-03	Genomics data & precision medicine	+20	Women	PhD in Molecular Biology from St. John’s University, New York Postdoctoral research at the University of Massachusetts, Worcester	Private	Engaged in genomics data research and precision medicine practice, contributing to the scientific and technical implementation of genomic testing
SME-04	Quality assessment of medicines	+15	Women	Master’s degree in Pharmaceutical Quality PhD in Business Administration	Government	Involved in the quality assessment of medicines, including regulatory compliance activities relevant to advanced therapy medicinal products.

SME, subject matter expert.

## Data Availability

Data supporting the finding of this study are contained within this article. The regulatory documents analyzed in this study are publicly available from the respective agencies (SFDA, MOH, FDA, EMA), and access information is provided in the References Section.

## Supplementary Materials

The following supporting information can be downloaded at: https://www.mdpi.com/article/10.3390/healthcare14183029/s1, Table S1: Completed COREQ (Consolidated Criteria for Reporting Qualitative Research) 32-Item Checklist for This Study.

## Abbreviations

The following abbreviations are used in this manuscript:

AI	artificial intelligence
ATMP	Advanced Therapy Medicinal Product
CAR	chimeric antigen receptor
EMA	European Medicines Agency
FDA	United States Food and Drug Administration
ICH	International Council for Harmonization of Technical Requirements for Registration of Pharmaceuticals for Human Use
KACST	King Abdulaziz City for Science and Technology
KAIMRC	King Abdullah International Medical Research Center
KAUST	King Abdullah University of Science and Technology
KFSHRC	King Faisal Specialist Hospital & Research Centre
MOH	Ministry of Health
NCBE	National Committee of Bioethics
NCC	National Cancer Center
NGH	National Guard Hospital
PDPL	Personal Data Protection Law
Saudi NIH	Saudi National Institute of Health
SDAIA	Saudi Data and Artificial Intelligence Authority
SFDA	Saudi Food and Drug Authority
SME	subject matter expert

## Appendix A

**Table A1:** Themes and key questions guiding the structured interview.

Theme	Key Questions
Section 1: Demographics and Background	Q1: Kindly introduce yourself with a brief description of your role and experience in using genomics data in personalized oncology medicine. Include the specific area you specialize in (e.g., medical oncology, research and ethics, genomics science, or data specialist) and the years of experience, your primary responsibilities regarding using genomics data and personalized medicine in your current work, and your role in interactions with regulatory agencies concerning genomic data and personalized medicine in oncology. Describe if you are involved in developing guidelines related to personalized oncology medicine. Q2: Based on your experience, how do you view the use of personalized medicine in cancer treatment?
Section 2: Current Practices and Implementation	Q1: What are the strategies and current practices in place for using genomics data in treating cancers in your setting? Describe the perspectives of healthcare providers, patients, and collaboration among your organization and relevant stakeholders in Saudi Arabia. Q2: What specific regulations or policies are currently in place regarding genomic data and personalized medicine according to personalized medicine implementation in cancer treatment, ethics, privacy, and data handling, healthcare providers’ training, and patient consent and education? Do you find these regulations sufficient, or do they require improvement? Q3: How common is using genomics data within the oncology practice in your setting and why? Q4: Can you share one example of a successful case where genomics data influenced cancer care outcomes by using advanced personalized medicine and another example of a failed case where it did not achieve the expected results?
Section 3: Challenges and Barriers	Q1: Based on your experience, what challenges arise in the use of genomic data and the advancement of personalized medicine in oncology, related to healthcare providers, patients, and collaboration among stakeholders? Q2: What are the challenges regarding your current regulations, policies, ethics, or data protection in genomics data and personalized oncology medicine? Q3: How do national regulatory requirements, such as those from the SFDA, MOH, or organizational standards, impact the use of genomics data? Q4: Are there any additional challenges you face that we have not covered?
Section 4: Recommendations and Solutions	Q1: From your perspective, do you think that incorporating genomic data into personalized oncology medicine is important or not? Why? Q2: What improvements or solutions do you believe could address the challenge discussed previously, according to personalized medicine implementation in cancer treatment, ethics, privacy, and data handling, healthcare providers’ training, and patient consent and education? Q3: How can stakeholders (e.g., regulatory bodies, healthcare providers, and researchers) collaborate to improve genomics integration? Q4: What strategies can be used to improve market access for utilizing genomics data in personalized oncology medicine? Q5: From your experience, do you have any additional recommendations you would like to share?
Section 5: Future Perspectives	Q1: What do you envision as the future role of genomics data in the oncology field in Saudi Arabia? Q2: What emerging advancements do you believe will shape the future of genomics and personalized medicine in oncology? Q3: Do you have any final thoughts or key takeaways on the future of genomics in personalized oncology?
