# Supplementary material for: Using Genomic Data in Personalized Oncology Medicine: An Exploratory Expert-Elicitation Study with Narrative Regulatory Mapping of Regulations, Implementations, and Challenges in Saudi Arabia
Source: Healthcare (Basel). 2026 Sep 15;14(18):3029. doi: 10.3390/healthcare14183029 (PMC13606128; doi:10.3390/healthcare14183029)
Supplement: Supplementary file 1 [file healthcare-14-03029-s001.zip › healthcare-4358596-supplementary.pdf]

COREQ (Consolidated Criteria for Reporting Qualitative Research) 32-Item Checklist

Completed for: "Using Genomic Data in Personalized Oncology Medicine: An Exploratory Expert-Elicitation Study with Narrative Regulatory Mapping of Regulations, Implementations, and Challenges in Saudi Arabia"

Lamees Hakami, Lamya Alnaim, Khalid Almoaikel, Jawaher Alharbi

Healthcare (MDPI) — Manuscript under revision

Section references correspond to the submitted manuscript. Items marked "Not reported" reflect genuine gaps acknowledged by the authors in the Limitations section.

No.	Domain & Item	Guide Question	Reported on Page/Section No.	Notes
DOMAIN 1: Research team and reflexivity				
Personal characteristics				
1	Interviewer/facilitato r	Who conducted the interview or focus group?	Not explicitly stated	The primary researcher (L.H.) conducted data analysis and is identified as the lead throughout the Methods section (Section 2.3.5). Although it is implied that L.H. led the data collection, the manuscript does not explicitly state who conducted the interviews. Future reporting should name the interviewer(s) directly.
2	Credentials	What were the researcher's credentials, e.g., PhD, MD?	Not reported	No academic credentials (e.g., degree, professional qualifications) are provided for L.H. or the other members of the research team beyond their institutional affiliations on the title page. This is a reporting gap.
3	Occupation	What was their occupation at the time of the study?	Title page (Author affiliations)	L.H. is identified as affiliated with IQVIA Middle East and North Africa, Riyadh, Saudi Arabia. L.A. is affiliated with Pharmacy College, King Saud University. K.A. is affiliated with IQVIA MENA. J.A. is affiliated with King Faisal Specialist Hospital and Research Center.
4	Gender	Was the researcher male or female?	Not reported	The gender of the interviewer/lead researcher (L.H.) is not disclosed in the manuscript. This information is relevant for reflexivity and not currently provided.
5	Experience and training	What experience or training did the researcher have?	Not reported	No information is provided regarding L.H.'s prior qualitative research training, experience with expert interviews, or familiarity with the topic area. A reflexivity or positionality statement would address

				this gap.
Relationship with participants				
6	Relationship established	Was a relationship established prior to study commencement?	Not reported	The manuscript does not describe any prior relationship between the researchers and the participants before the study began. The co-author (K.A.) nominated the participants, which implies an indirect prior relationship, but this is not described in terms of its implications for the research.
7	Participant knowledge of the interviewer	What did participants know about the researcher? e.g., personal goals, reasons for doing the research	Not reported	No information is provided about what was disclosed to participants regarding the researcher's identity, background, study purpose, or personal interests in the topic prior to interviews.
8	Interviewer characteristics	What characteristics were reported about the interviewer/facilitator? e.g., bias, assumptions, reasons and interests in the research topic	Not reported	No reflexivity statement, positionality disclosure, or discussion of potential researcher bias, assumptions, or interests is included. The manuscript acknowledges single-coder bias in the Limitations (Section 4) but does not provide a broader reflexive account of the interviewer's characteristics or how these may have influenced data collection and interpretation.
DOMAIN 2: Study design				
Theoretical framework				
9	Methodological orientation and theory	What methodological orientation was stated to underpin the study? e.g., grounded theory, discourse analysis, ethnography, phenomenology, content analysis	Methods, Sections 2.1, 2.2, and 2.3.5	A mixed-methods exploratory design is stated in Section 2.2. The qualitative strand employs inductive thematic analysis, with six steps described in Section 2.3.5 (familiarisation, initial coding, theme development, refinement, triangulation, reporting). NVivo 14 was used for coding. No single overarching theoretical paradigm (e.g., constructivism, interpretivism) is explicitly named; the methodological orientation is described procedurally rather than epistemologically.
Participant selection				
10	Sampling	How were participants selected? e.g., purposive, convenience, consecutive, snowball	Methods, Section 2.3.2	Purposive/convenience sampling by nomination was used. Participants were nominated by co-author Dr. Khalid Almoaikel based on his familiarity with experts in the field. The manuscript acknowledges this introduces potential nomination bias and limits representativeness (Section 2.3.2). Selection criteria included ≥10 years of professional experience and representation from government and

				private sectors.
11	Method of approach	How were participants approached? e.g., face-to-face, telephone, mail, email	Not reported	The manuscript does not describe the mechanism by which participants were initially contacted or invited to participate (e.g., by email, telephone, or personal introduction). This is a reporting gap.
12	Sample size	How many participants were in the study?	Abstract; Methods, Section 2.3.2; Results, Section 3.3	Four subject-matter experts (n = 4) participated. Five were initially invited; four consented. The manuscript acknowledges the small sample size as a limitation and cautions against generalisability (Section 2.3.2 and Section 4).
13	Non-participation	How many people refused to participate or dropped out? Reasons?	Methods, Section 2.3.2	One of five invited individuals declined to participate. No reason for refusal is reported in the manuscript.
Setting				
14	Setting of data collection	Where was the data collected? e.g., home, clinic, workplace	Methods, Section 2.3.1	Interviews were conducted in person and online via Microsoft Teams and Zoom (Section 2.3.1). Interviews took place in February 2025. No further description of the physical setting (e.g., office, hospital meeting room) is provided for the in-person interviews.
15	Presence of non-participants	Was anyone else present besides the participants and researchers?	Not reported	The manuscript does not address whether any third parties (e.g., colleagues, observers, note-takers) were present during the interviews.
16	Description of sample	What are the important characteristics of the sample? e.g., demographic data, date	Results, Table 2 (Section 3.3)	Comprehensive demographic characteristics are presented in Table 2, including Expert ID (SME-01 to SME-04), area of specialisation, years of experience, sex, education level (degrees and institutions), employment sector (government/private), and role in genomic policy or oncology implementation.
Data collection				
17	Interview guide	Were questions, prompts, guides provided by the authors? Was it pilot tested?	Methods, Section 2.3.4; Appendix A, Table A1	A structured interview guide comprising 19 questions organised across five themes (current practices, challenges, barriers, solutions, and future recommendations) is described in Section 2.3.4 and reproduced in full in Table A1 (Appendix A). Pilot testing of the guide is not reported.
18	Repeat interviews	Were repeat interviews carried out? If yes, how many?	Not reported	The manuscript does not mention whether participants were interviewed more than once. All references suggest a single interview per participant.
19	Audio/visual recording	Did the research use audio or visual recording to collect the data?	Methods, Section 2.3.1	Each interview was individually audio-recorded (Section 2.3.1). Recordings were subsequently transcribed and organised

				according to key themes. No video recording is mentioned.
20	Field notes	Were field notes made during and/or after the interview or focus group?	Not reported	The manuscript does not mention the use of field notes during or after the interviews. This is a reporting gap; it is unclear whether observational or contextual notes were taken alongside audio recording.
21	Duration	What was the duration of the interviews or focus group?	Methods, Section 2.3.3	Each qualitative structured interview lasted approximately 45 minutes, with one exception that lasted 2 hours because the expert provided particularly comprehensive information (Section 2.3.3). This range is clearly reported.
22	Data saturation	Was data saturation discussed?	Not reported	Data saturation is not discussed in the manuscript. Given the intentionally small purposive sample (n = 4) in an acknowledged exploratory study, saturation was likely not the sampling criterion; however, this should be explicitly addressed in reporting.
23	Transcripts returned	Were transcripts returned to participants for comment and/or correction?	Not reported (Limitation acknowledged in Discussion, Section 4)	Member-checking was not conducted. The manuscript explicitly acknowledges this as a limitation: "no member-checking was conducted with participants to validate theme interpretation" (Section 4, Limitations). This is an honest and transparent disclosure.
DOMAIN 3: Analysis and findings				
Data analysis				
24	Number of data coders	How many data coders coded the data?	Discussion/Limitations, Section 4	Coding was conducted by a single researcher (L.H.), as explicitly acknowledged in the Limitations section: "coding was conducted by a single researcher, which may introduce coder bias — inter-rater reliability was not assessed" (Section 4). The manuscript recommends future studies employ multiple coders and establish inter-rater reliability (e.g., Cohen's kappa > 0.8).
25	Description of the coding tree	Did authors provide a description of the coding tree?	Methods, Section 2.3.5 (partial)	The thematic analysis process is described in six sequential steps (familiarisation, initial coding, theme development, refinement, triangulation, and reporting) in Section 2.3.5. A preliminary coding framework was built from the interview guide themes. However, a formal visual coding tree, node hierarchy, or code-to-theme mapping table is not presented; the description is procedural rather than structural.
26	Derivation of themes	Were themes identified in	Methods, Section	A hybrid approach was used. An a priori

		advance or derived from the data?	2.3.5; Results, Section 3.2	coding framework was first developed based on the five interview guide themes (Section 2.3.5, Step 2). Themes were then refined inductively through iterative comparison across transcripts. The six final themes are presented in Section 3.2. The initial deductive structure was supplemented by inductive theme refinement.
27	Software	What software, if applicable, was used to manage the data?	Methods, Section 2.3.5	NVivo software (version 14, QSR International) was used for line-by-line coding of interview transcripts (Section 2.3.5). Manual review was also used to supplement software-assisted coding to ensure contextual accuracy, as noted in the Limitations (Section 4).
28	Participant checking	Did participants provide feedback on the findings?	Not reported (Limitation acknowledged in Discussion, Section 4)	Member-checking was not performed. The manuscript explicitly discloses this as a limitation in Section 4: "no member-checking was conducted with participants to validate theme interpretation." This is transparent; the authors recommend it for future studies.
Reporting				
29	Quotations presented	Were participant quotations presented to illustrate the themes/findings?	Not reported	The manuscript does not include direct verbatim quotations from participants. Expert views are summarised and attributed to specific SME IDs (e.g., SME-01, SME-02, SME-03, SME-04) throughout the Results section, but no in-text direct quotes are provided. Inclusion of representative direct quotations would strengthen qualitative rigour in future revisions.
30	Data and findings consistent	Was there consistency between the data presented and the findings?	Results, Sections 3.3–3.8; Discussion, Section 4	The findings presented in Sections 3.3–3.8 are internally consistent with the stated six themes (Section 3.2) and the Discussion (Section 4). Expert-attributed insights are triangulated with published literature and regulatory documents throughout. Quantitative context notes are added under several subsections to clarify the boundaries of expert-based claims.
31	Clarity of major themes	Were major themes clearly presented in the findings?	Results, Section 3.2; Sections 3.3–3.8	Six primary themes are clearly listed in the Thematic Analysis Overview (Section 3.2): (1) Demographics and Background, (2) Current Practices and Implementation, (3) Challenges and Barriers, (4) Recommendations and Solutions, (5) Future Perspectives, and (6) Regulatory Comparison. Each theme is addressed in a

				dedicated subsection (Sections 3.3–3.8) with further sub-themes, providing a clear thematic structure.
32	Clarity of minor themes	Is there a description of diverse cases or discussion of minor themes?	Results, Sections 3.4–3.6 (partial)	Sub-themes are identified within each major theme and addressed in numbered subsections (e.g., 3.4.1 Healthcare Providers, 3.4.2 Patients, 3.4.3 Collaboration). However, a formal description of minor, negative, or deviant cases is not included, which is consistent with the exploratory design. Individual expert views are attributed by SME IDs but divergent or minority views among experts are not explicitly discussed.

Abbreviations

COREQ: Consolidated Criteria for Reporting Qualitative Research

L.H.: Lamees Hakami (primary researcher and lead analyst)

SME: Subject-Matter Expert

NVivo 14: Qualitative data analysis software (QSR International, Version 14)

IQVIA: An American multinational company focused on clinical research services and health information technology

SFDA: Saudi Food and Drug Authority

SDAIA: Saudi Data and Artificial Intelligence Authority

KACST: King Abdulaziz City for Science and Technology

KFSHRC: King Faisal Specialist Hospital and Research Center

NCBE: National Committee of Bioethics

ATMP: Advanced Therapy Medicinal Products

Reference[30]. : Tong A, Sainsbury P, Craig J. Consolidated criteria for reporting qualitative research (COREQ): a 32-item checklist for interviews and focus groups. Int J Qual Health Care. 2007;19(6):349–357. https://doi.org/10.1093/intqhc/mzm042
